# Massive drainage from incision after tunica vaginalis eversion: A case of patent processus vaginalis

**DOI:** 10.1016/j.eucr.2025.103275

**Published:** 2025-11-11

**Authors:** Wenbin Bao, Xiaodong Hu, Xuebing Ma, Yangwen-Yi Liu, Jinghong Duan, Ren Huang

**Affiliations:** a Department of Urology, The Fourth Affiliated Hospital of Dali University, Chuxiong, 675000, China; b Department of Urology, Chuxiong People's Hospital, Chuxiong, 675000, China

**Keywords:** Hydrocelectomy complication, Processus vaginalis patency, Hemodialysis ascites scrotal leakage

## Abstract

A 68-year-old male on hemodialysis developed torrential incisional leakage (2000–3000 mL/day) 5 days after left testicular tunica vaginalis eversion. Fluid analysis confirmed ascites. Imaging revealed a patent processus vaginalis communicating with the incision. Conservative management with inguinal compression dressing and dialysis optimization achieved complete resolution within 72 hours. Follow-up at 2 months showed no recurrence. This represents the first documented hemodialysis-associated communicating hydrocele leakage. Successful closure resulted from: 1) pressure gradient reversal collapsing the processus vaginalis, and 2) restored lymphatic equilibrium (drainage > ascites production) without surgical intervention.

## 1 Introduction

Massive incisional fluid leakage following testicular tunica vaginalis eversion is a rare occurrence. We report a case involving hemodialysis-associated ascites leaking through a latent patent processus vaginalis communicating with the surgical incision.

## 2 Case report

**Fig. 1:**
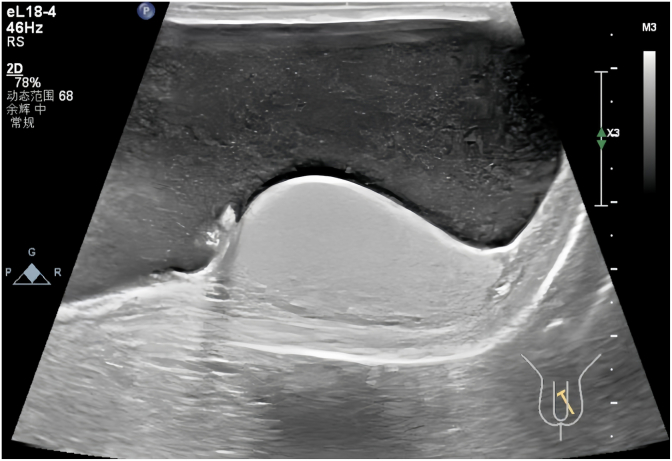
Scrotal ultrasound Ultrasound indicates bilateral testicular hydroceles, with the left side being more prominent.

**Fig. 2:**
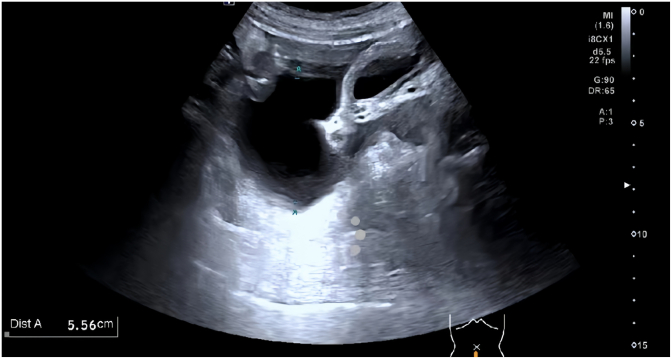
Pelvic and abdominal fluid collections (maximal depth ∼5.5 cm).

**Fig. 3:**
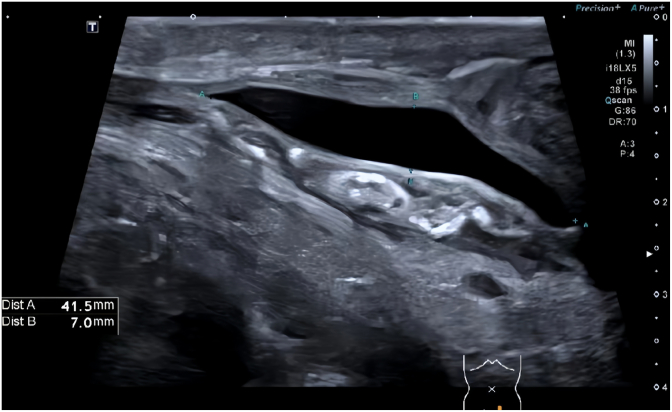
A band-like anechoic tract (approximately 4.1 × 0.7 cm) along the left spermatic cord in the inguinal region.

A 68-year-old male presented with persistent left scrotal enlargement for over six months. Scrotal ultrasound confirmed bilateral testicular hydroceles, larger on the left (Fig. 1). On April 6, 2025, he underwent left hydrocelectomy. On postoperative day 5, the patient developed copious leakage of clear yellow fluid from the incision, with intermittent jet-like discharge totaling 2000–3000 mL daily. Physical examination revealed mild left inguinal swelling, fluid leakage from the left scrotal incision, and left testicular enlargement without significant tenderness. Repeat pelvic and scrotal ultrasound demonstrated:Pelvic and abdominal fluid collections (maximal depth ∼5.5 cm) (Fig. 2). A band-like anechoic tract (approximately 4.1 × 0.7 cm) along the left spermatic cord in the inguinal region (Fig. 3). This structure had well-defined margins, good sound transmission, extended superiorly towards the inguinal canal (suggesting communication with the peritoneal cavity), and connected inferiorly to the surgical incision. A linear hypoechoic focus (1.0 × 0.48 cm) adjacent to the incision. Color Doppler flow imaging (CDFI) showed no flow within these structures. Fluid analysis results were:Red Blood Cells: +++, Total Protein: 32.8 g/L,Albumin: 18.8 g/L (↓), Globulin: 14.0 g/L (↓), Lactate Dehydrogenase: 101 U/L,Triglycerides: 0.48 mmol/L (↓), Urine Creatinine: 98 μmol/L.

Based on the biochemical profile (low protein, low triglycerides, low urine creatinine), Sonographic findings definitively demonstrate a patent processus vaginalis, and the patient's history of end-stage renal disease (ESRD) on long-term hemodialysis, the leakage was attributed to peritoneal fluid extravasation through a patent processus vaginalis communicating with the incision. Conservative management with a left inguinal compression dressing was initiated, resulting in gradual resolution of the leakage. No recurrence was observed during a 2-month follow-up period.

## 3 Discussion

Preoperative assessment (ultrasound, non-reducible, non-fluctuant scrotal mass unaffected by position) supported the diagnosis of testicular hydrocele, leading to tunica vaginalis eversion. Common complications of scrotal surgery include hematoma, infection, and recurrent/persistent hydrocele.^1^ Massive postoperative incisional leakage is highly unusual. Initial suspicion for a urinary fistula was discounted by biochemical fluid analysis. Notably, this patient had ESRD managed by hemodialysis. While communicating hydroceles and dialysate leaks are well-documented complications in continuous ambulatory peritoneal dialysis (CAPD) patients (incidence: 7–27.5 %; rate: 0.04–0.08 per patient-year).^2^^,^^3^ In a previously reported similar case^4^,a 67-year-old male with ESRD managed by CAPD for one year, presented with acute-onset right testicular edema following nonproductive coughing episodes. Abdominopelvic CT peritoneography showed contrast tracking from the peritoneal cavity through the right inguinal canal into the scrotum. Similar findings have also been reported by Estalella et al.^5^ But, this mechanism is unexpected in hemodialysis patients. Communicating hydroceles arise from congenital patency of the processus vaginalis. Symptoms may manifest in childhood or adulthood, often triggered by increased intra-abdominal pressure. Under physiological conditions, minimal fluid passage through a small patent processus vaginalis is counterbalanced by active fluid absorption from the tunica vaginalis, preventing hydrocele development. However, during peritoneal dialysis—where significant intra-abdominal pressure increases occur—voluminous fluid transit through the patent processus vaginalis disrupts this dynamic equilibrium, leading to symptomatic hydroceles. Critically, these symptoms arise from hydrodynamic alterations in response to elevated intra-abdominal pressure, rather than from recanalization of a closed processus vaginalis. It is important to distinguish this pathophysiology from that of acquired non-communicating hydroceles, which are well-documented and typically result from inflammation, trauma, or infection. Fundamentally, increased intra-abdominal pressure acts as a trigger for converting anatomically patent but clinically silent processus vaginalis into symptomatic communicating hydroceles.

In this hemodialysis patient, two synergistic mechanisms explain the massive postoperative leakage:

1. Ascites exacerbation: First, inadequate fluid removal during hemodialysis or inaccurately set dry weight leading to systemic fluid retention; second, worsening heart failure exacerbating sodium and water retention within the body.
2. Surgically Disrupted Equilibrium:Postoperatively, surgical disruption of the tunica vaginalis created a low-resistance conduit, permitting the extravasation of ascitic fluid *via* the patent processus vaginalis. We propose this patient had preexisting subclinical communicating hydrocele. The narrow patency of the processus vaginalis would have allowed only gradual fluid accumulation within the tunica vaginalis. This insidious process—compounded by chronically elevated intra-abdominal pressure—masked classic signs of communicating hydrocele.

The patient received comprehensive management including optimized hemodialysis, heart failure correction, nutritional support, and left inguinal compression dressing:a gauze bolster secured with elastic bandage, changed every 24–48 hours for 10 days. Following this regimen, scrotal incisional leakage resolved without recurrence during 2-month follow-up. The therapeutic efficacy is mediated through two synergistic pathways: firstly, reversal of pressure gradients achieved by compression dressing application, which elevates tissue pressure to levels exceeding intra-abdominal pressure, thereby mechanically collapsing the patent processus vaginalis; secondly, restoration of dynamic lymphatic equilibrium via dual mechanisms—reduction in ascites production through optimized hemodialysis and improved cardiac decongestion, coupled with enhancement of lymphatic drainage capacity secondary to systemic edema resolution, establishing a state where lymph flow exceeds the ascites generation rate. Notably, while isolated HD-related ascites is uncommon, significant ascites typically requires synergistic factors like heart failure - highlighting our patient's specific risk profile. This integrated approach reestablishes physiological fluid homeostasis through functional closure of the pathological communication channel rather than anatomical obliteration. Furthermore, this case underscores that a meticulous intraoperative search for a patent processus vaginalis is imperative in all hydrocelectomies to prevent this specific complication.

## 4 Conclusion

This case establishes hemodialysis-associated fluid overload and ascites as a previously unrecognized precipitant for symptomatic communicating hydrocele, a complication previously associated primarily with peritoneal dialysis. It critically illustrates how surgical alteration of local hydrodynamics can transform a latent anatomical variant (patent processus vaginalis) into a major complication pathway for ascites drainage. Therefore, a communicating hydrocele cannot be definitively excluded in adult patients presenting with hydroceles, particularly those with conditions predisposing to ascites or elevated intra-abdominal pressure. This diagnostic possibility warrants active consideration during preoperative evaluation. Concurrently, meticulous intraoperative inspection for patency of the processus vaginalis is imperative, regardless of preoperative findings, to prevent such unexpected complications.

## CRediT authorship contribution statement

**Wenbin Bao:** Writing – original draft, Investigation, Conceptualization. **Xiaodong Hu:** Visualization, Formal analysis, Data curation. **Xuebing Ma:** Validation, Resources, Methodology. **Yangwen-Yi Liu:** Writing – review & editing, Resources. **Jinghong Duan:** Investigation, Data curation. **Ren Huang:** Writing – review & editing, Supervision, Project administration.
